# Clonally unrelated HL-type RS manifested as hemophagocytic syndrome: a case report and literature review

**DOI:** 10.3389/fonc.2024.1472560

**Published:** 2024-12-05

**Authors:** Xiaoman Zheng, Wenshuang Ding, Zhigang Zhu, Junping Li, Weijie Zhong

**Affiliations:** ^1^ Department of Geriatrics, Hematology & Oncology Ward, Guangzhou First People’s Hospital, Guangdong Medical University, Guangzhou, Guangdong, China; ^2^ Department of Pathology, Guangzhou First People’s Hospital, Guangdong Medical University, Guangzhou, Guangdong, China

**Keywords:** Hodgkin lymphoma variant of Richter syndrome, chronic lymphocytic leukemia, small lymphocytic lymphoma, hemophagocytic syndrome, clonal relationship

## Abstract

Hodgkin lymphoma variant of Richter syndrome (HL-type RS) is a very rare disease, in which chronic lymphocytic leukemia (CLL) or small lymphocytic lymphoma (SLL) is transformed into novel Hodgkin lymphoma. The most important prognostic factor of HL-type RS is the clonal relationship between HL-type RS and the preexisting CLL/SLL. Detailed confirmation of clonally unrelated HL-type RS cases have not been reported. To the best of our knowledge, this is the first case of HL-type RS confirmed as clone independent by a detailed comparison of immunoglobulin gene rearrangement clones and gene mutations. A 76-year-old man, diagnosed with SLL 1 year before transformation was treated with Zanubrutinib 3 months before transformation. When diagnosed with HL-type RS, he presented with symptoms of hemophagocytic syndrome. Positive therapeutic effects were achieved using a modified rituximab-doxorubicin, bleomycin, vinblastine, dacarbazine regimen in combination with Zanubrutinib. We also discuss a thorough review of the relevant literature we performed to help us better understand this rare disease.

## 1 Introduction

Richter syndrome (RS) is defined as the development of an aggressive lymphoma in patients with chronic lymphocytic leukemia (CLL) or small lymphocytic lymphoma (SLL). The majority of cases transform into diffuse large B-cell lymphoma (DLBCL), with the remainder transforming into Hodgkin lymphoma (HL) or other aggressive lymphomas, known as the DLBCL variant (DLBCL-type RS) and the HL variant (HL-type RS), respectively (1, 2). The survival rates of the two types of RS differ markedly, with a median overall survival (OS) of 5.9 months for DLBCL-type RS and 30.8 months for HL-type RS. The prognosis of HL-type RS is significantly better (3), but the median OS of HL-type RS was still significantly lower than that of primary classical HL (4). HL-type RS is rare, and the most important prognostic factor of HL-type RS is the clonal relationship between HL-type RS and the preexisting CLL/SLL. The clonally related HL-type RS has a significantly worse prognosis, while the clonally unrelated HL-type RS has a relatively better prognosis (5).

Currently, few cases of HL-type RS have been reported, no case of confirmed clonally unrelated HL-type RS has been reported, and there are also a lack of literature reviews on HL-type RS. Here, we present a recent case of clonally unrelated HL-type RS, which was confirmed by PCR and a large panel of next-generation sequencing (NGS). The patient experienced a good outcome. A detailed review of relevant literature was performed and is discussed.

## 2 Case presentation

**Figure 1:**
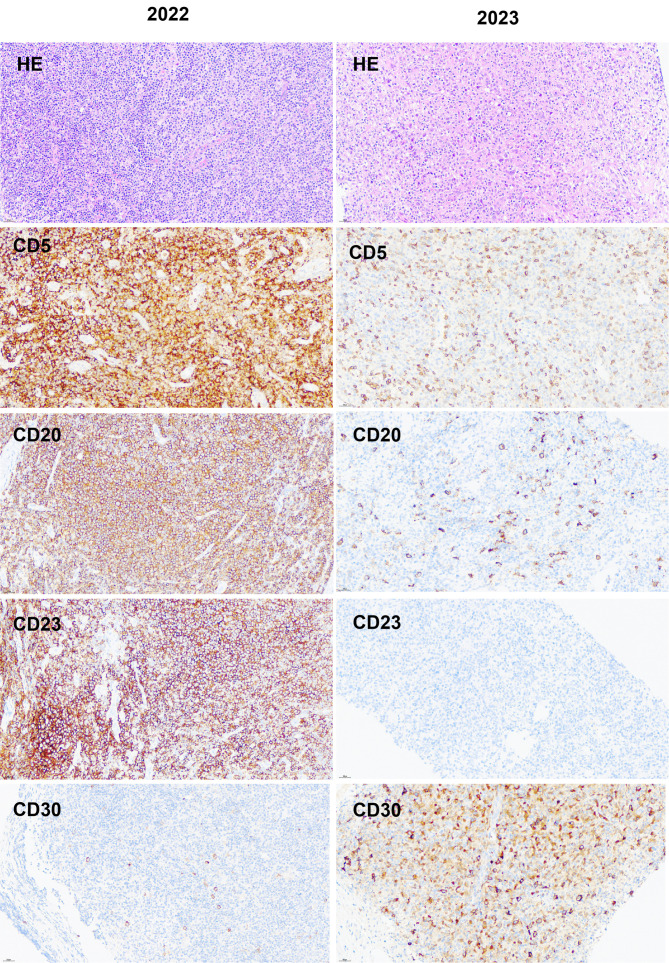
Comparison of immunohistochemical images from two lymph node paraffin tissues.

A 76-year-old man was admitted to our hospital department on May 19, 2022, due to recurring abdominal pain for 5 days, with no fever, night sweats, or weight loss. He had a past medical history of coronary heart disease, hypertension, and was a hepatitis B virus carrier. An enlarged lymph node with an approximate size of 4 × 2.5 cm was palpated in the right inguinal region. Laboratory results were presented in **Table 1**. An enhanced computed tomography (CT) scan of the whole abdomen revealed multiple enlarged and fused lymph nodes in the abdominal cavity, retroperitoneum, pelvic cavity, right inguinal region, and right diaphragmatic angle area. The largest was located in the hepatogastric gap and measured approximately 5.3 × 3.0cm. Complete excision biopsy of the right inguinal lymph node was performed on May 31, 2022. The pathological result was showed in **Table 1** (**Figure 1**). Whole-body positron emission tomography (PET)/CT result was also showed in **Table 1**. Bone marrow cytology and biopsy showed no bone marrow invasion. The bone marrow karyotype analysis was 46 XY, inv (9) (p11q13)c (20). The NGS (222 genes) results of lymph node paraffin tissue are shown in **Table 2**. The 222 genes panel included UNC13D gene, but no other familial hemophagocytic lymphohistiocytosis related genes. The clonal analysis results of immunoglobulin (Ig) gene rearrangement are shown in **Table 3**. Combined with the results of PET/CT, blood routine, lymph node pathology, and bone marrow examination, the diagnosis was SLL (Lugano stage IIIA). The patient did not meet the indications of initiating treatment and was instructed to be followed up in the clinic.

**Table 1:** Results of laboratory workup.

Date	Workup	Results
May, 2022	Blood routine tests	White blood cell count (WBC) 4.72×10^9^/L, absolute lymphocyte count (LYM) 1.00×10^9^/L, absolute neutrophil count (NEUT) 3.14×10^9^/L, hemoglobin (HGB) 133g/L, total platelet count (PLT) 210 × 10^9^/L.
	Other laboratory results	Lactate dehydrogenase (LDH) 198 U/L and hepatitis B viral DNA level < 100 IU/mL.
	The pathological result of the right inguinal lymph node	The pathological diagnosis was CLL/SLL. Immunohistochemistry (**Figure 1**) revealed that the tumor cells were positive for CD20, CD79a, PAX5, CD5, CD23, MUM-1 (partially), and BCL-2, background T cells were positive CD3, some helper T cells were positive for CD4, BCL6, PD-1, and CXCL13, some T cells were positive for CD8, with approximately 40% of cells positive for Ki-67.
	Whole-body PET/CT	Numerous enlarged lymph nodes both above and below the diaphragm. The maximum size was 4.0 × 2.4 cm. The maximum standardized uptake value (SUVmax) was 6.9 and the average SUV (SUVave) was 3.5.
February, 2023	Blood routine tests	WBC 2.16×10^9^/L, LYM 0.29×10^9^/L, NEUT 1.38×10^9^/L, HGB 80 g/L, and PLT 114×10^9^/L.
May, 2023	Blood routine tests	WBC 1.01×10^9^/L, LYM 0.12×10^9^/L, NEUT 0.41×10^9^/L, HGB 83g/L, PLT 114×10^9^/L, and LDH 256 U/L.
	Peripheral blood pathogenic microorganisms NGS	It detected 4741 sequences of Epstein-Barr virus (EBV).
	Other laboratory results	Peripheral blood EBV DNA 3.49×10^4^ copies/mL, peripheral blood interferon-gamma 22.4 pg/mL, interleukin (IL)-6 294.71 pg/mL, IL-10 85.61 pg/mL, ferritin 3350 ng/mL, triglycerides 1.77 mmol/L, soluble IL-2 receptor (sCD25) 24183 U/mL (normal reference range 223-710 U/mL), and natural killer cell activity 13.25% (normal reference range ≥15.11%).
	Whole-body PET/CT	Several enlarged lymph nodes with increased metabolism were discovered in many lymph node areas both above and below the diaphragm, with a maximum size of approximately 5.9×4.6 cm, SUVmax 12.6, and SUVave 9.3. The spleen was enlarged, occupying approximately 8 rib units (**Figure 2**).
June, 2023	The pathological result of the enlarged lymph node in the left axilla	The pathological diagnosis was the transformation from CLL/SLL to HL. Immunohistochemistry (**Figure 1**) revealed that Hodgkin-like cells were positive for CD30, CD20, CD79a, PAX-5 (weak), MUM-1, C-MYC, BCL6 (partially), and BCL-2; Ki-67 was approximately 60%; and EBV-encoded RNA was positive.

**Table 2:** Comparison of two NGS (222 genes) results from lymph node paraffin tissue.

No.	gene	chromosome	Transcript number	exon	Nucleotide changes	amino acids changes	variant allele frequency
**1**	*CTNNB1*	3p22.1	NM_001904.4	5	c.521T>C	p.Met174Thr	1.50%
	*ID3*	1p36.12	NM_002167.5	1	c.190C>T	p.Leu64Phe	2.60%
	*IGLL5*	22q11.22	NM_001178126.2	1	c.68G>A	p.Arg23His	18.40%
	*MAP2K1*	15q22.31	NM_002755.3	2	c.170A>C	p.Lys57Thr	27.10%
**2**	*TET2*	4q24	NM_001127208.2	3	c.2746C>T	p.Gln916Ter	2.30%
	*TET2*	4q24	NM_001127208.2	3	c.2887C>T	p.Gln963Ter	6.60%
	*FAS*	10q23.31	NM_ 000043.6	8	c.6761G>C	p.?	4.80%
	*IGLL5*	22q11.22	NM_001178126.2	1	c.188T>A	p. Leu63Gln	2.00%
	*IGLL5*	22q11.22	NM_001178126.2	1	c.153_154delCCinsTT	p. Pro52Ser	2.40%
	*VPREB1*	22q11.22	NM_ 007128.4	2	c.113G>A	p.Arg38His	55.30%

1 means "Results when diagnosed with SLL".

2 means "Results when diagnosed with HL-RS".

**Table 3:** Comparison of two immunoglobulin gene rearrangement results from lymph node paraffin tissue.

No.	Gene rearrangement type	Cloning results
**1**	IgH(FR1/FR2/FR3-JH)	Positive (+)
	IgK(Vκ-Jκ/Kde)	Positive (+)
	IgL(Vλ-Jλ)	Negative (−)
**2**	IgH(FR1/FR2/FR3-JH)	Positive (+)
	IgK(Vκ-Jκ/Kde)	Negative (−)
	IgL(Vλ-Jλ)	Negative (−)

1 means "Results when diagnosed with SLL".

2 means "Results when diagnosed with HL-RS".

On February 20, 2023, the patient was readmitted to our hospital due to recurrent fever for 3 days, and multiple blood routine tests showed progressive decrease in whole blood cells (**Table 1**). Repeated CT plain scans of the chest and whole abdomen suggested multiple lymph nodes were enlarged compared to the previous ones. Re-examination of bone marrow cytology and immunophenotyping showed no significant abnormal population. We considered that the patient’s SLL had progressed and he met the indication for initiating treatment. Oral administration of Zanubrutinib capsules 160mg bid was initiated on March 3, 2023. Within the first three months of taking Zanubrutinib, the superficial enlarged lymph nodes had shrunk and the blood routine tests had basically returned to normal.

**Figure 2:**
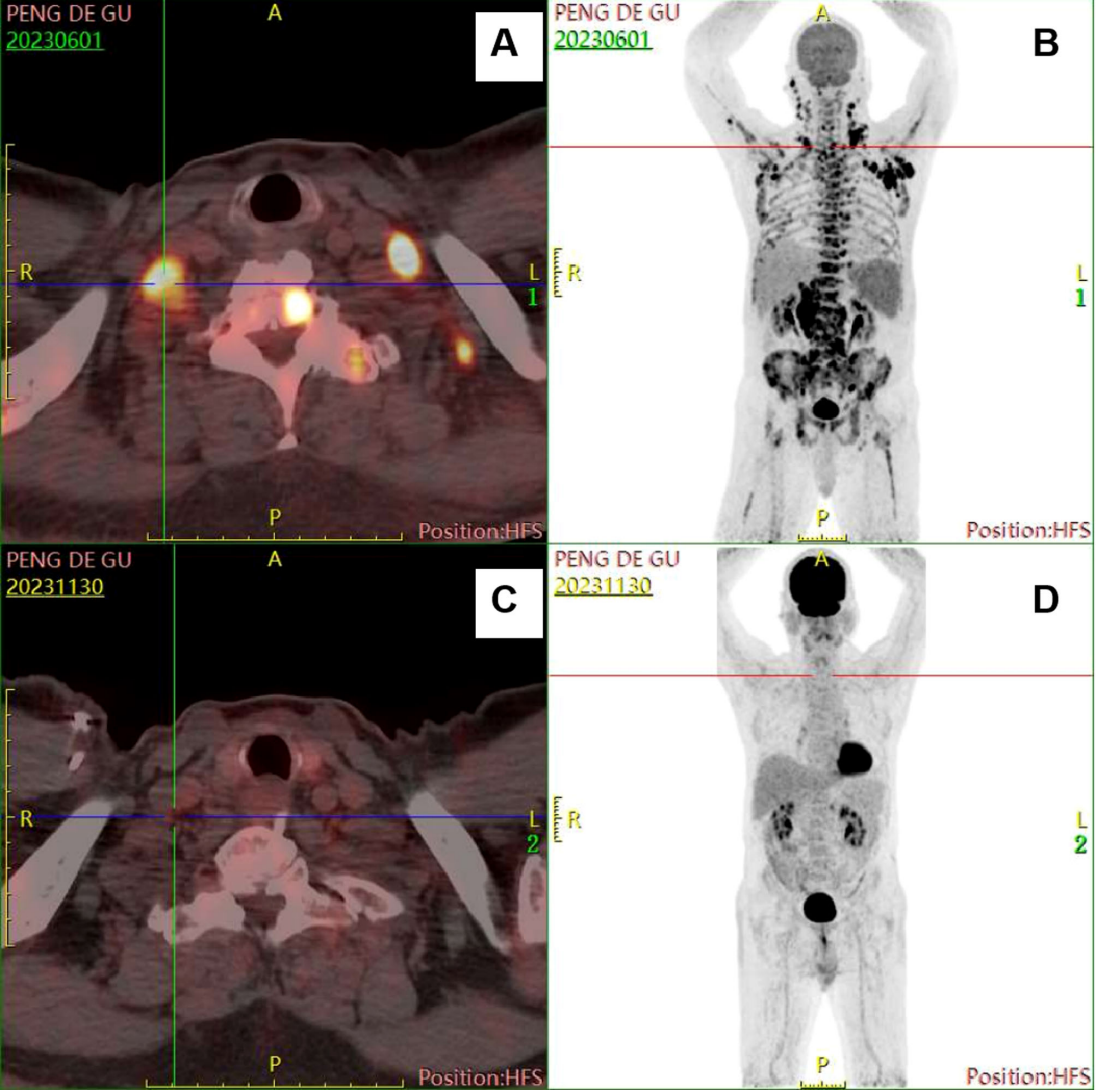
Comparison of images from two PET/CT examinations. **(A, B)** images when diagnosed with HL-type RS. **(C, D)** images at the end of chemotherapy treatment.

However, on May 25, 2023, the patient was admitted to our hospital again due to repeated high fever for 1 week, with persistent high fever and a maximum body temperature of 39-40°C, requiring the use of antipyretic drugs to reduce the fever. Laboratory results were presented in **Table 1**. No hemophagocytic cells were found in bone marrow cell morphology and immunophenotyping examinations. Whole-body PET/CT result was showed in **Table 1** (**Figure 2**). According to the 2004 diagnostic criteria for hemophagocytic lymphohistiocytosis (HLH), the patient met 6 of 8 criteria, and the diagnosis of HLH was clear. Dexamethasone, ruxolitinib, and human immunoglobulin were immediately administered due to the patient’s critical condition.

On June 5, 2023, a biopsy was performed on the enlarged lymph node in the left axilla. The pathological result was showed in **Table 1** (**Figure 1**). The results of NGS (222 genes) of lymph node puncture tissue are shown in **Table 2**. The results of Ig gene rearrangement clone analysis are shown in **Table 3**. Based on the significant increase in LDH, symptoms, PET-CT and new pathological results, the diagnosis was that SLL had transformed into HL (stage IIIB, IPS 5 points), namely RS, accompanied by EBV infection.

**Figure 3:**
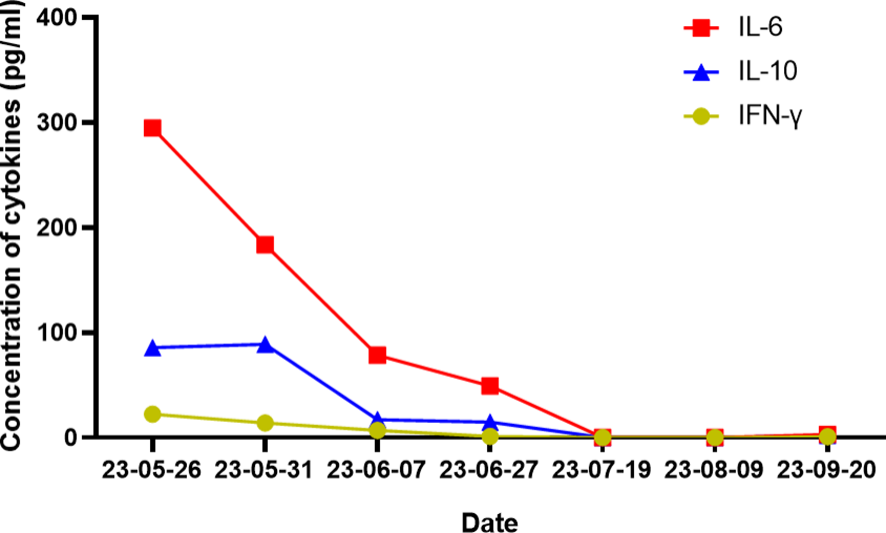
Images of changes in multiple cytokines.

According to the results presented in **Tables 2**, **3**, the Ig gene rearrangements and mutated genes detected in the two tests were completely unrelated, indicating that the transformed HL was clonally unrelated to the primary SLL. Due to the second pathological results indicating CD20 positive and the patient’s advanced age of 76 years, an 8-course modified rituximab-doxorubicin, bleomycin, vinblastine, dacarbazine (R-ABVD) chemotherapy regimen (rituximab 600mg intravenous infusion d0, doxorubicin liposomes 40 mg intravenous infusion d1, bleomycin 15,000 units intravenous infusion d1, vincristine 3 mg intravenous infusion d1, and dacarbazine injection 500 mg intravenous infusion d1 every 21 days). After one course of chemotherapy, HLH-related symptoms were significantly relieved, and the patient no longer had a fever. The HLH therapeutic efficacy reached complete remission (CR) after 3 courses of chemotherapy. The changes in the peripheral blood cytokine levels are shown in **Figure 3**. The therapeutic effect was evaluated to be CR by whole-body enhanced CT scan after 4 courses of chemotherapy. After 8 courses of chemotherapy, whole-body PET/CT indicated that a complete metabolic remission was achieved (**Figure 2**). The patient is currently maintaining a state of sustained CR. Maintenance therapy of Zanubrutinib 160mg bid was continued during and after chemotherapy.

## 3 Discussion and literature review

RS, also known as Richter’s Transformation, is a term defined as a specific form of CLL/SLL transforming into aggressive lymphoma, with an incidence rate of approximately 2-10% yearly (6, 7). More than 90% of RS cases transform to DLBCL, with the remainder transforming to HL and other rare forms of lymphomas (8). The incidence of HL-type RS in CLL/SLL population was 0.4-0.7%, accounting for less than 5% of RS cases (9), and the annual incidence rate is 0.05% (8).

HL-type RS is rare in clinical conditions. Up to now, only approximately 100 cases of HL-type RS have been reported in the international literature (8, 9). while no more than 5 cases have been reported in China (10). Among these 5 cases, none were tested for clonal relation before and after transformation, or for clonal relation only by IgH rearrangements through PCR. So, these 5 cases have obvious limitations. Moreover, there are very few literature reviews or reviews on HL-type RS. This elderly patient is the first reported case of clone independent HL-type RS, confirmed by detailed PCR and a big panel of NGS (222 genes). We promptly diagnosed HLH and HL-type RS and administered a modified R-ABVD regimen combined with Zanubrutinib, which achieved a very good outcome. As of the time of publication, the patient has been followed up for more than 15 months starting from the diagnosis of RS and is currently in a continuous CR state.

HL-type RS is more common in the elderly, mainly in males (male-to-female ratio approximately 3:1 to 6:1) (11), with a median age of 64-72 years at the time of HL-type RS diagnosis (8, 9, 11–14) and a median time from CLL/SLL to HL-type RS diagnosis of 3.2-7.5 years (9, 11–14). The common clinical symptoms of HL-type RS include lymph node enlargement, B symptoms, fever, splenomegaly, hepatomegaly, fatigue, and night sweats (13). Nearly half (47%) of HL-type RS patients experience elevated LDH levels (13), 82.9-87% of patients are clinically staged Ann Arbor stage III-IV, and 65% of patients have an International Prognostic Score > 4 (11, 15). Patients with HL-type RS often have EBV infection, with a reported EBV positivity rate of 67% (11), and EBV positivity rate in pathological sections of up to 71% (16). This case was also an elderly male, who was 76 years old when he was diagnosed with HL-type RS. The interval from SLL to HL-type RS progression was only about 1 year, which was significantly shorter than the average level reported in the literature. The patient’s symptoms at the time of transformation were fever, pancytopenia, enlarged lymph nodes, B symptoms, splenomegaly, and markedly elevated LDH level, combined with EBV infection. The diagnosis was HLH. Chaker et al. also reported an EBV positive case of HL-type RS presenting as HLH (17). Therefore, it is important to identify and diagnose HLH promptly, while also being vigilant for the occurrence of RS.

The clonal correlation before and after transformation is crucial for the treatment and prognosis of RS. So far, the clonal correlation between DLBCL-type RS and CLL/SLL has been fully elucidated. A previous study found through NGS that mutations in *TP53*, *NOTCH1*, *MYC*, and *CDKN2A* genes were closely associated with 90% of DLBCL-type RS cases (1). Moreover, a recent study confirmed through single-cell sequencing that the MYC BACH2/JUNB/RGS2, and IFR9/PRDX1 pathways, amino acid metabolism, proteasome pathway, and immune escape are all involved in the occurrence and development of DLBCL-type RS (18). However, the clonal correlation between HL-type RS and CLL/SLL has rarely been reported, and only a few studies have analyzed the clonal correlation between HL-type RS and CLL/SLL by detecting the IgH rearrangement or IgHV mutation rate by PCR. So, the clonal correlation between HL-type RS and CLL/SLL, and the biological characteristics of HL-type RS are still unknown (1, 14). Dujardin et al. detected IgH rearrangements by PCR and found that 7 of 12 HL-type RS patients had clonal correlation with pre-transformation CLL/SLL, who were identical to classical RS and had a poor prognosis. The other 5 HL-type RS patients had no clonal correlation with pre-transformation CLL/SLL, and these patients were usually EBV positive and had a better prognosis (5). Mauro et al. also used PCR and fluorescence *in situ* hybridization techniques and found that 18 of 28 HL-type RS patients had no IgHV mutations, 13 of 25 had no chromosomal abnormalities, 6 of 25 had 13q-, 6/25 had +12, 1/25 had 17p-, and 1of 14 had TP53 mutations (14). Xiao et al. used two methods, laser capture microdissection and PCR, to demonstrate that the expression of ZAP-70 in CLL cells from pathological sections correlated with the clonal correlation of HL-type RS, whereas EBV status or morphological patterns were not associated with the clonal correlation (16). HL-type RS transformed from ZAP-70-positive CLL was not related to CLL clones, whereas HL-type RS transformed from ZAP-70-negative CLL was related to CLL clones, and rarely could clonally-independent HL-type RS cells be derived from ZAP-70-negative CLL (16, 19). For the first time in our case of HL-type RS, detailed comparison of Ig gene rearrangement clonal and gene mutation analyses were performed in pathological tissues before and after transformation, using both PCR and NGS in a large panel. Before transformation, IgH and IgK rearrangement clones were positive, IgL rearrangement clone was negative, and *CTNNB1*, *ID3*, *IGLL5*, and *MAP2K1* gene mutations were detected. After transformation, IgH rearrangement clone was positivity, IgK and IgL rearrangement clones were negative, and *TET2*, *FAS*, *IGLL5*, and *VPREB1* gene mutations were detected (**Tables 1**, **2**). It can be clearly seen from the results that the HL-type RS in this case was not related to the SLL clone before transformation, and that this patient harbored an EBV infection and had a good outcome after chemotherapy, consistent with a report in the literature (5). Detailed Ig gene rearrangement clonal analysis and gene mutations data before and after the transformation of this patient will enrich the data on the biological characteristics of HL-type RS.

Therapeutic advances in RS in recent years have mainly focused on DLBCL-type RS. Ongoing clinical research continues to explore new therapies or drugs including chimeric antigen receptor-T therapy, bispecific antibodies, Bruton tyrosine kinase inhibitors, Programmed cell death protein 1/Programmed death-ligand 1 inhibitors, Venetoclax, and antibody coupled drugs, with some breakthroughs realized (20, 21). However, relatively few new drugs can be used for HL-type RS. Given the extremely low prevalence of HL-type RS, clinical trials of new drugs are impossible (1). Because of similarities with primary HL, HL-type RS was usually treated with ABVD or modified ABVD chemotherapy regimens as the first-line regimens, with treatment response rates of 40-60% and median OS of approximately 4 years for HL-type RS (8, 9, 13, 15). A recent study, which is one of the largest single-center real-world population-based cohort studies to date (11), found that 32 patients with HL-type RS had a median OS of 31 months, with 2-year progression-free survival (PFS)and OS of 47% and 57%, respectively. The median OS was significantly higher than the previously reported median OS of 8-9.4 months for DLBCL-type RS (22, 23), but lower than the median OS for primary HL. The 5-year OS of advanced primary HL has reached 87% (4). However, patients who were able to tolerate chemotherapy with standard ABVD or modified ABVD regimens showed an improved 2-year PFS and OS of 70% and 74%, respectively, which was similar to the 2-year OS of elderly primary HL patients reported by the same researchers earlier (24). However, the median age of the cohort population was as high as 71 years, and more than one-third of the patients could not tolerate the ABVD regimen. As a result, the survival of the entire population was significantly worse than that of primary HL. Another multicenter study from the United States, with the largest number of HL-type RS patients to date, reported a total of 94 patients enrolled in the study, with a median OS of up to 65 months and a 2-year OS of 72%. The patients who were able to tolerate the standard ABVD-based therapy had a median OS of up to 13.2 years, far exceeding the median OS of the entire HL-type RS population. This may be due to the higher proportion of people aged <65 years who could tolerate the ABVD regimen in this study (25). The collective evidence confirms that the ABVD regimen is the standard first-line regimen for well-tolerated HL-type RS patients. The main possible adverse event of this regimen is severe pulmonary toxicity caused by exposure to bleomycin. If mid-term PET/CT reveals remission and no significant changes, bleomycin can be removed and the AVD regimen used after two cycles of the ABVD regimen (26). Concerning new drugs, both PD1 inhibitors and Brentuximab vedotin (an anti-CD30 monoclonal antibody coupled to monomethyl auristatin E as an ADC) have proven effective in primary refractory relapsed (R/R) HL, but there is insufficient data regarding their use in R/R HL-type RS (27, 28). Brentuximab vedotin in combination with AVD (A+AVD) has been used in the first-line treatment of advanced primary HL with superior results to the ABVD regimen (29). A recent case report applied A+AVD for the first-line treatment of a case of HL-type RS with good results (30). Another case of R/R HL-type RS resistant to Bendamustine, which progressed and subsequently received sequential treatment with Brentuximab vedotin and Pembrolizumab, with encouraging results (31). Based on these findings, PD1 inhibitors and Brentuximab vedotin might become important choices for the first- or second-line treatment of Hodgkin HL-type RS in the future. Given the older onset age of HL-type RS, very few patients could undergo hematopoietic stem cell transplantation (HSCT), and there is still a lack of evidence to support the role of HSCT. In the largest cohort of HL-type RS cases reported to date, only 7 of 94 patients received HSCT after the first CR, but their 2-year OS was not significantly better than that of HL-type RS patients who did not receive HSCT (25).

In this case, although the patient was diagnosed with RS at the age of 76-years-of-age, we chose a modified R-ABVD regimen based on the pathological results, which prolonged the chemotherapy intervals of the ABVD regimen as well as reducing the doses of various drugs and the total number of cycles, in combination with Zanubrutinib to control SLL. The treatment achieved very satisfactory results.

Up to now, after more than 15 months of follow-up, the patient is still in CR, similar to the aforementioned results from the literature. In the future, if the disease progresses, PD1 inhibitors and Brentuximab vedotin will be the second-line treatment choice.

## Data Availability

The original contributions presented in the study are included in the article/supplementary material, further inquiries can be directed to the corresponding author/s.
